# Atypical manifestations of cardiomegaly and nephrotic syndrome in Kawasaki disease: Two case reports

**DOI:** 10.1097/MD.0000000000018117

**Published:** 2019-11-27

**Authors:** Liyuan Wang, Xiaomei Sun, Xiaotang Cai, Shunli Liu, Zhiling Wang, Yongmei Xie

**Affiliations:** a Department of Pediatrics, West China Second University Hospital; b Key Laboratory of Obstetric & Gynaecologic and Pediatric Diseases and Birth Defects of Ministry of Education, Sichuan University, Chengdu, 610041, Sichuan, China.

**Keywords:** cardiomegaly, Kawasaki disease, pericardial effusion

## Abstract

**Rationale:**

To examine atypical manifestations of Kawasaki disease (KD) in children.

**Background:**

Cardiovascular complications during acute KD are a major contributor to its mortality rate. It can involve the pericardium, the myocardium, the endocardium, and/or the coronary arteries; however, cardiomegaly and nephrotic syndrome (NS) during the acute stage of KD have seldom been reported.

**Patient concerns:**

Two children, each with a fever lasting more than 5 days, were diagnosed with cardiomegaly using echocardiography in the early phase of Kawasaki disease (within 2 weeks). Case 1 was misdiagnosed with NS because of the proteinuria, hypoalbuminemia, and edema present at the onset of the disease.

**Diagnoses:**

A diagnosis of incomplete KD was based on a constellation of clinical manifestations and symptoms and was supported by laboratory results.

**Interventions:**

Intravenous immunoglobulin (IVIG) and aspirin were administered, supplemented with and without supplemental steroid therapy (case dependent).

**Outcomes:**

The clinical manifestations and syndromes of the two cases were completely resolved and their heart size restored to normal within 2 weeks, with no evidence of coronary artery lesions (CAL).

**Main lessons:**

Physical findings and manifestations are atypical in incomplete KD. Cardiomegaly and nephrotic syndrome can be an early manifestation of KD; cardiomegaly, especially, should be recognized as a possible manifestation of the acute stage of KD. Furthermore, these symptoms can be rapidly relieved by treatment with IVIG, with or without supplemental steroid therapy.

## 1 Introduction

Kawasaki disease (KD) is an acute, febrile, systemic vasculitis of unknown etiology that predominantly affects children <5 years of age.^[1]^ Although it affects individuals from all races, it is most common in Asian populations, especially those with Japanese ancestry. The average annual incidence rate of KD in Japan is 264.8 per 100,000 children under 5 years of age, compared to only 11.4, 5.4, and 7.4 per 100,000 in Finland, Norway, Sweden, respectively, and 20.8 in USA.^[2–4]^ KD can damage multiple organs; induce coronary artery lesions (CAL); and/or cause carditis, hepatitis, arthritis, central nervous system disease, KD shock syndrome, muscle and kidney damage, and hyponatremia.^[1]^ Cardiovascular complications during acute KD are a major contributor to its mortality rate. It can involve the pericardium, the myocardium, the endocardium, and/or the coronary arteries; however, cardiomegaly and nephrotic syndrome (NS) during the acute stage of KD have seldom been reported. Here, we report cases of two Chinese children with cardiomegaly in the acute stage of KD, one of whom experienced concomitant NS at the onset of the disease.

This study retrospectively analyzed the clinical data of 2 children with KD who were admitted between 2016 and 2017 in the West China Second University Hospital and followed up for 6 months. This study was approved by the Ethics Committee of the West China Second University Hospital. Patients’ parents have provided informed consent for publication of the case.

A total of 2 children with cardiomegaly in early phase of Kawasaki disease are included in this series.

## 2 Case 1

**Figure 1:**
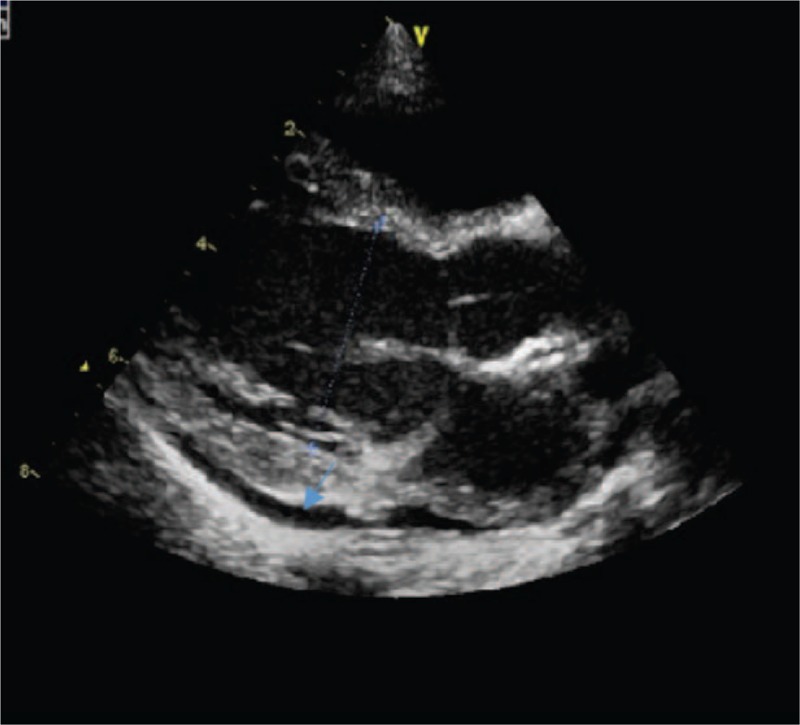
Echocardiography showed a small amount of pericardial effusion (PE).

**Figure 2:**
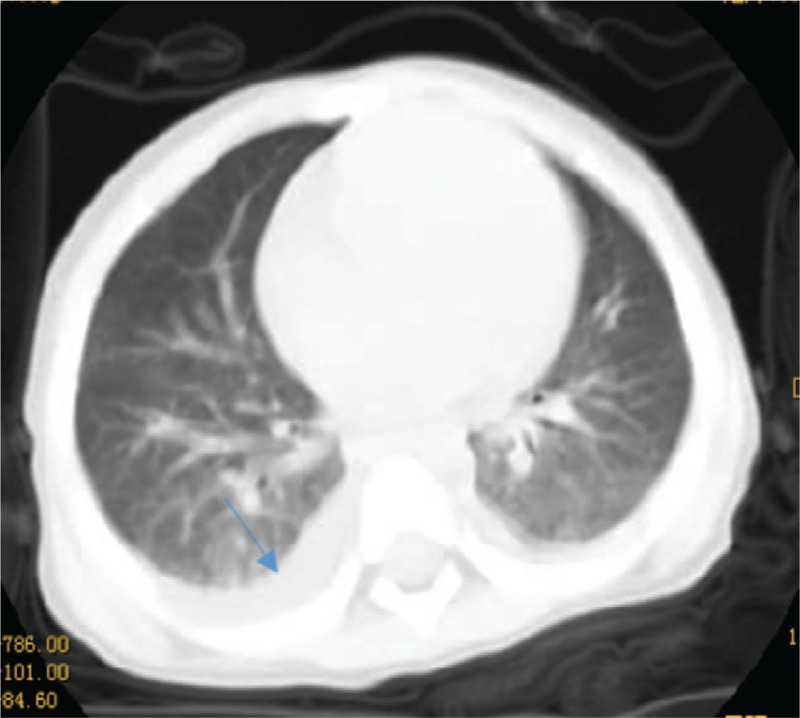
The CT of the chest showed blurred patches and streaked shadows in the lower regions of the lungs with small bilateral pleural effusions.

A 23-month-old boy was admitted to our hospital for high fever lasting 4 days with abdominal distention and mild edema of the limbs for 1 day; his highest temperature was 39.1 °C and was accompanied by cough and chills. He received cefoperazone and tazobactam for 2 days in a local hospital, but fever did not abate and symptoms of edema, oliguria, and poor appetite developed, with a rash covering a portion of his trunk and extremities. Laboratory tests at a local hospital were as follows: white blood cell count (WBC) 1.85 × 10^9^/L (3.6–9.7 × 10^9^/L), percent of neutrophil granulocyte (N%) 66% (23.6–75%), hemoglobin (Hb) 104 g/dL (110–146 g/L), platelet count (PLT) 187 × 10^9^/L (100–450 × 10^9^/L), C-reactive protein (CRP) 62.54 mg/L (0–8 mg/L), triglyceride (TG) 3.81 mmol/L (<2.83 mmol/L), serum potassium 2.93 mmol/L (3.5–5.5 mmol/L), serum sodium 138.7 mmol/L (135–145 mmol/L). Urine test: protein was 2+, red blood cell (RBC) and pathocast were negative. Urine and blood cultures were sterile. The physical examination on admission revealed a temperature of 38.6 °C, a respiration rate of 25 beats/min, a heart rate of 114 beats/min, a blood pressure of 99/68 mm Hg, and mildly irritated, red skin rashes over the body with normal skin between. The eyelids and toes were edematous. The bilateral conjunctiva had hyperaemia without tumefaction of the lymph node. Additionally, the patient displayed cracked lips, a strawberry tongue, perianal desquamation, and abnormalities at the Bacilli Calmette-Guerin inoculation site. The abdomen was distended (abdominal perimeter was 49 cm) and positive for shifting dullness. The physical examination of the lung, heart, and nervous systems was normal. The laboratory evaluation of our hospital on admission was as follows: WBC 5.4 × 10^9^/L, N% 69.3%, Hb 101 g/L, PLT 152 × 10^9^/L, CRP 40.0 mg/L (0–8 mg/L), erythrocyte sedimentation rate (ESR) 2 mm/hour (<21 mm/hour), alanine aminotransferase (ALT) 55 U/L (21–72 U/L), aspartate aminotransferase (AST) 67 U/L (17–59 U/L), lactic dehydrogenase (LDH) 994 U/L (313–618 U/L), albumin 24.7 g/L (35–50 g/L), serum sodium 131.9 mmol/L (137–145 mmol/L), serum potassium 3.5 mmol/L (3.5–5.1 mmol/L), and serum calcium 2.01 mmol/L (2.1–2.55 mmol/L). Routine urine: protein 2+, RBC 0–3/hp, WBC 0–3/hp, pathocast 1+. 24-hours proteinuria: 0.5 g/24 h (<0.14 g/24 h). Urine and blood cultures were sterile. Coagulation function test: International Normalized Ratio (INR) 1.16 (0.8–1.5), prothrombin time (PT) 14.3 seconds (9.4–15.4 seconds), activated partial thromboplastin time (APTT) 60.0 seconds (20.6–40.6 seconds), fibrinogen (Fg) 166 mg/dL (200–400 mg/dL), D-dimer 6.11 mg/L (<0.55 mg/L), fibrinogen degradation product (FDP) 20.9 μg/mL (<5 μg/mL). The assessment of immune function: IgG 23.90 g/L (3.41–19.6 g/L), IgA 0.64 g/L (0.19–2.20 g/L), IgM 0.94 g/L (0.43–1.63 g/L), complement C3 0.42 g/L (0.70–2.06 g/L), complement C4 0.27 g/L (0.11–0.61 g/L), CD3 73.9 (39–73%), CD4 46.7 (25–50%), CD8 24.1 (11–32%), CD4/CD8 1.9 (1.5–2.0). Echocardiography showed that the left heart was slightly enlarged (left ventricle (LV) = 36 mm, left atrium (LA) = 22 mm) with normal heart function (ejection fraction (EF) = 62%, patent foramen ovale, normal size of the coronary arteries (CA), (left coronary artery (LCA) = 2.3 mm, *Z*-score <2; left anterior descending coronary artery (LAD) = 2.4 mm, *Z*-score <2; left circumflex coronary artery (LCX) = 1.2 mm, *Z*-score <2; right coronary artery (RCA) = 2.0 mm, *Z*-score <2), and a small amount of pericardial effusions (PEs) (Fig. 1). A CT of the chest showed blurred patches and streaked shadows in the lower regions of the lungs with small bilateral pleural effusions (Fig. 2). The CT of the abdomen showed effusion and pneumatosis in the small intestine, slight dilation in some of the intestinal tubes, and effusion in the abdomen and pelvic cavity.

After admission, he was diagnosed with incomplete KD and treated with immunoglobulin (2 g/kg). His abdominal distension and limb edema abated, and his temperature normalized. One day later (3rd day of hospitalization), his fever returned. After immunoglobulin (1 g/kg) was administered, followed by aspirin (50 mg/kg/day), his temperature returned to normal. On the next day (5th day of hospitalization), the echocardiograph indicated that the left heart was less enlarged than before (LV = 32 mm, LA = 23 mm) and without CAL. Routine urine and 24-hours proteinuria tests returned to normal 3 days later (8th day of hospitalization), and the aspirin dosage was reduced to 5 mg/kg/day. Within 4 days, his temperature normalized, and the patient was discharged (12th day of hospitalization). One week after discharge, the WBC was 5.1 × 10^9^/L, the Hb rose to 108 g/L, and the PLT increased to 543 × 10^9^/L. CRP levels returned to normal. Two weeks after discharge, echocardiography revealed that the size of the heart returned to normal without evidence of CAL. The child underwent routine echocardiography, blood tests, and urine tests for 1 year; all follow-up tests were normal.

## 3 Case 2

**Figure 3:**
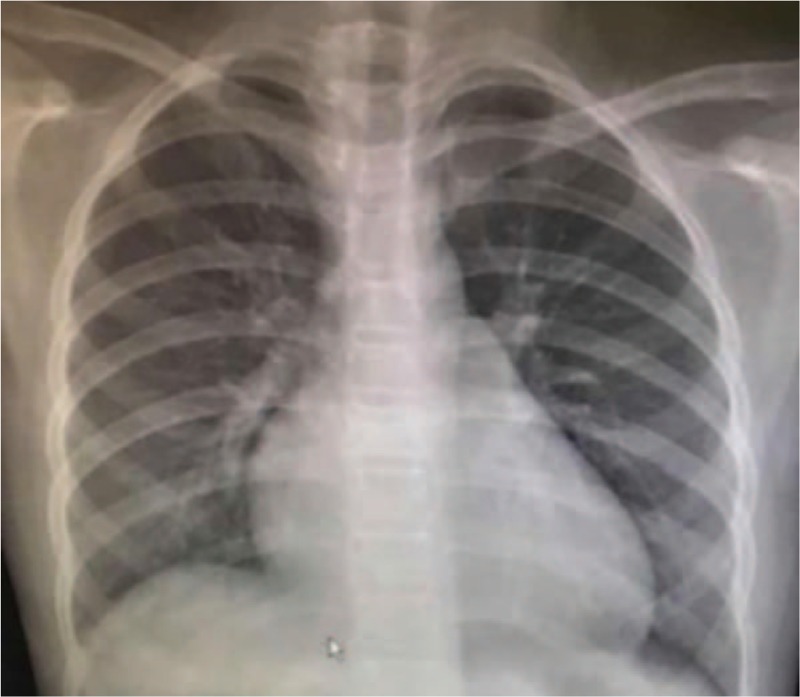
Chest radiography found heart shadow enlargement, and the cardiothoracic ratio was 0.62.

**Figure 4:**
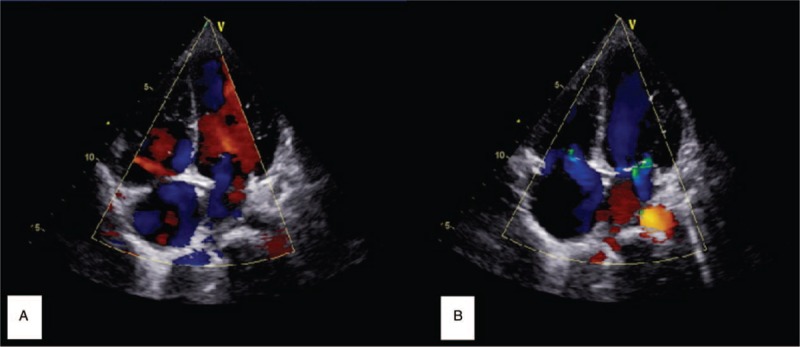
Echocardiography showed slight or moderate mitral regurgitation in the mitral valve (A) and in the tricuspid valve (B).

A 10 years and 5-month-old girl was admitted to our hospital for fever for the last 8 days with recent discovery of cardiomegaly. Her highest temperature was 39.2 °C and was accompanied by right submandibular neck lymph node enlargement, abdominal discomfort, and rash. Although she was treated with antibacterial and antiviral drugs for several days in the previous hospital, she still had a high temperature, vomiting, and sickness. In addition, chest radiography found a heart shadow enlargement (Fig. 3) and a blood test showed her troponin level was increased to 0.396 ng/mL (0.00–0.066 ng/mL). The girl was referred to our hospital for a suspected diagnosis of myocarditis. The physical examination of admission revealed a body temperature of 37.7 °C, a respiration rate of 21 beats/min, a heart rate of 90 beats/min, and a blood pressure of 92/50 mm Hg. She presented with a normal level of consciousness. The skin on her lower limbs and trunk displayed a scattered, red, polymorphic rash, which faded with pressure and did not involve more than the skin surface. The right submandibular lymph nodes were 3 cm × 2 cm and slightly tender. Hemorrhagic foci (2 mm × 3 mm) were seen in the left bulbar conjunctiva. The physical examination of the other systems was otherwise unremarkable. The laboratory data were as follows: WBC 26.0 × 10^9^/L, N% 82%, Hb 98 g/L, PLT 387 × 10^9^/L, CRP 103 mg/L, ESR 73 mm/h, ALT 194 U/L, AST 314 U/L, albumin 29.2 g/L, LDH 1130 U/L, troponin 0.156 μg/L and CK-Mb was normal. Coagulation function test: INR 1.3, PT 16.0 seconds, APTT 36.1 seconds, Fg 469 mg/dL, TT 16.4 seconds. The test of urine routine was normal. Thyroid function: T_3_ 1.00 nmol/L (1.4–3.7 nmol/L), T4 87.7 nmol/L (82–171 nmol/L), TSH 3.615 mIU/L (0.64–6.27 mIU/L), anti-thyroglobulin antibody 104.6 μ/mL (<60 U/mL) and anti-thyroperoxidase antibody 206.7 U/mL (<60 U/mL). Autoantibodies tests showed antinuclear antibodies (ANA) were positive (1:100). Echocardiography showed a slightly enlarged heart (LV = 45 mm, LA = 28 mm, RV = 16 mm, RA = 52 mm) (Fig. 4), slight or moderate mitral regurgitation in the mitral valve (*V*_max_ = 4.9 m/second) and in tricuspid valve (*V*_max_ = 2.4 m/second), normal heart function (EF = 62%), and normal sized coronary arteries (*Z*-score <2). Enhanced chest CT test showed a little shadow on the upper lobe of the lungs and stripes on the lower lobe of the left lung, a few pleural effusions, and a slightly enlarged heart shadow.

After admission, she was treated with intravenous immunoglobulin (2 g/kg) for the diagnosis of incomplete KD. Her temperature returned to normal and the erythema disappeared after 2 days. We added a small dose of prednisone (0.5 mg/kg day) and aspirin (3 mg/kg day) for successive treatment. Three days after intravenous immunoglobulin (IVIG), we rechecked the echocardiography, which revealed that the cardiac size was slightly recovered (LV = 43 mm, LA = 28 mm, RV = 20 mm, RA = 48 mm), and the heart function was still normal. One week after IVIG, echocardiography showed a normal cardiac size (LV = 40 mm, LA = 22 mm, RV = 10 mm, RA = 38 mm) without CAL. A reexamination of the blood revealed WBC 7.6 × 10^9^/L, N% 62.7%, HGB 117 g/L, PLT 526, CRP 8 mg/L. After discharge, the patient underwent routine echocardiography and blood test for 6 months; all follow-up tests were normal.

## 4 Discussion

These two cases were diagnosed with incomplete KD in accordance with the 2017 guideline of KD from the American Heart Association.^[1]^ The patient in case 1 had a fever for 7 days; 3 principal clinical features (rash, edema of the hands and feet, and bilateral bulbar conjunctival injection); and 4 laboratory findings (CRP >3.0 mg/dL, platelet count of >450,000 after the 2 weeks of fever, albumin <3.0 g/dL, anemia). The patient in case 2 had fever for 10 days; only 2 principal clinical features (rash and cervical lymphadenopathy); but 5 laboratory findings (significantly elevated WBC count, anemia, platelet count of >450,000 after the 2 weeks of fever, albumin <3.0 g/dL, and an elevated ALT level). Echocardiography in both patients revealed early phase cardiomegaly, which was completely relieved after IVIG was administered with or without steroid therapy.

An estimated 15–20% of KD patients are diagnosed with incomplete KD, and the incidence rate is thought to be rising over the years.^[5,6]^ Young infants (especially aged <6 months) and older children are more likely to experience incomplete KD.^[7,8]^ Although the prognosis of children with incomplete KD is comparable to those with complete KD, prolonged fever is one of the strongest predictors of CAL.^[9]^ Compared with typical KD, the physical findings of incomplete KD usually lack 2 of the 6 basic symptoms, most frequently cervical lymphadenopathy and polymorphous exanthema; however, characteristic mucous membrane changes are present in both typical and incomplete disease.^[10]^ On the other hand, incomplete KD can present with atypical manifestations, such as pneumonia, nephritis, arthritis, uveitis, myositis, retinal vasculitis, and central nervous system involvement,^[11]^ and NS and cardiomegaly are both rare clinical manifestations that can appear during the acute stage of KD. One case in our article was diagnosed with NS because of proteinuria, hypoalbuminemia, and edema; cardiomegaly was apparent in both patients in this report.

Noncoronary cardiac abnormalities, including PE, valvar regurgitation, left ventricular dysfunction, and aortic root dilation are common in the acute stage of KD^[12,13]^; however, cardiomegaly has rarely been reported and the pathogenic mechanism is still unclear. The patient in case 1 had PE with normal levels of CK-Mb and troponin and lacked evidence of myocarditis, but vasculitis was evident because of the kidney involvement. As Okada^[14]^ reported, vasculitis and microvascular hyperpermeability are important events in the pathophysiology of pericardial effusion in KD. The patient in case 2 had mitral regurgitation combined with an increased level of both CK-Mb and troponin, which suggested that the cardiomegaly might be caused by myocarditis. Suzuki^[15]^ also reported that tricuspid and mitral regurgitation are often transient in the acute phase of KD and could be attributed to myocarditis. Crystal^[16]^ suggested that ventricular dilation may have subclinical myocardial involvement; it may be increased in patients on the initial echocardiography but remain within the normal range. Hence, cardiomegaly is a previously unrealized, type of cardiac damage involved in the acute phase of KD, whose etiology may be multi-factorial, involving vasculitis and myocarditis, and should be further studied. Cardiomegaly is not in the current guideline as a diagnostic manifestation; however, we need to be aware of the potential for this type of noncoronary damage when a diagnosis of incomplete KD is being considered.

KD is a systemic vasculitis that can result in kidney injures, including sterile pyuria, acute nephritis syndrome, renal tubular abnormalities, NS, and renal artery lesions (aneurysms and stenosis) that are seldom seen in patients.^[17]^ Only three articles, including 5 children, have reported NS in the acute phase of KD.^[18–20]^ In 1985, Lee reported a 3-month-old infant who presented with NS during the acute phase of KD and improved after steroid therapy. Unfortunately, the child died because of a massive myocardial infarction due to coronary aneurysm.^[18]^ Four other patients (aged 4, 4.5, 8 and 4.5 years) recovered completely without any sequelae, and the proteinuria in all of the patients disappeared within 2 weeks with administration of IVIG without steroids, similar to our patient in case 1. The pathogenic mechanism of NS in KD is still unclear, but activation of the immune system (such as T-cell immune-complex mediated kidney injury) is postulated as a possible mechanism.^[17,21,22]^

The patients’ clinical manifestations resolved and their hearts returned to a normal size after treatment with IVIG and aspirin, with or without steroid therapy. The patient in case 2 experienced a strong inflammatory response evidenced by high levels of CRP, ESR, WBC, and multiple positive antibodies; so low dose prednisone (1 mg kg^−1^ day^−1^) was administered for 1 week and then tapered down in a second week. Although the patient in case 1 had PE and cardiomegaly, IVIG was administered without corticosteroids because his inflammation biomarkers were not very high and his ESR and WBC were normal. Echocardiography revealed that both patients’ hearts had recovered within 1 month. According to the 2017 guideline of KD, corticosteroids were recommended in children at high risk for the development of coronary artery aneurysms.^[1,23]^ Whether PE is a risk factor for the development of CAL is debatable^[14]^; so the indications for steroid utilization in KD with noncoronary cardiac abnormalities are undefined.

## 5 Conclusion

Because of the increased rate of cases with incomplete KD, we are seeing more evidence of atypical manifestations. Cardiomegaly and nephrotic syndrome can be an early manifestation of KD; cardiomegaly, especially, should be recognized as a possible heart manifestation in the acute stage of KD. Furthermore, these symptoms are rapidly relieved by treatment with IVIG, with or without supplemental steroid therapy.

## Acknowledgments

The authors thank the patient's parents for providing permission to use the information of their children.

## Author contributions

**Conceptualization:** Xiaotang Cai.

**Data curation:** Liyuan Wang, Shunli Liu.

**Investigation:** Yongmei Xie.

**Methodology:** Zhiling Wang.

**Project administration:** Xiaotang Cai.

**Resources:** Xiaomei Sun.

**Supervision:** Zhiling Wang.

**Writing – original draft:** Liyuan Wang.

**Writing – review & editing:** Liyuan Wang, Xiaomei Sun, Xiaotang Cai.
